# Oncologic Outcomes and Safety of Neoadjuvant Treatment with Anthracyclines Versus Anthracycline-Free Regimens in HER2-Positive Early Breast Cancer in a Colombian Cancer Center: An Observational, Analytical, Retrospective Study

**DOI:** 10.3390/cancers17193190

**Published:** 2025-09-30

**Authors:** Alfredo Acevedo-Ramos, Andrea Zuluaga-Liberato, Sandra E. Díaz-Casas

**Affiliations:** 1 Medical Oncology Fellowship Program, Instituto Nacional de Cancerología, Universidad El Bosque, Bogotá 111511, Colombia; 2 Department of Medical Oncology, Instituto Nacional de Cancerología, Bogotá 111511, Colombia; azuluaga@cancer.gov.co; 3 Department of Breast and Soft Tissue Tumors, Instituto Nacional de Cancerología, Bogotá 111511, Colombia; sdiaz@cancer.gov.co

**Keywords:** breast cancer, HER2-positive, neoadjuvant, anthracyclines, pathologic complete response

## Abstract

**Background**: There are no comparative trials between the two most common schemes in HER2-positive early breast cancer treatment; BERENICE (with anthracyclines) and TRAIN-2 (without anthracyclines). In this study, we investigated the pathological complete response (pCR) and safety events achieved with each. **Methods**: This analytical retrospective observational study included 111 patients with early and locally advanced HER-2-positive breast cancer who initiated neoadjuvant treatment with an anthracycline-based scheme (four cycles of doxorubicin and cyclophosphamide, followed by four cycles of taxane, trastuzumab, and pertuzumab = AC-THP) and a non-anthracycline scheme (carboplatin, weekly paclitaxel, trastuzumab, and pertuzumab for six–nine cycles = TCbHP) at the National Cancer Institute in Colombia, between April 2020 and December 2024. The primary endpoint was the pCR. Safety was analyzed in patients who received at least one treatment cycle. **Results**: A total of 51 patients received AC-THP and 60 TCbHP (89.6% of which received six cycles). The pCR was 58.3% in ACHTP and 60.4% in TCbHP (*p* = 0.84). As a descriptive analysis, with the anthracycline-based scheme, there was a trend toward a higher pCR in patients with T3-T4, positive nodal involvement (N+), and positive hormone receptor (HR+). Cardiac toxicity events during the neoadjuvant phase were 9.8% in ACTHP and 3.3% in TCbHP. Grade 2 neuropathy events were higher in patients with the TCbHP scheme, at 23.3%, versus 9.8% in ACTHP. **Conclusions**: We found similar pCR rates between the schemes with anthracyclines and without anthracyclines. It is still pertinent to discuss the risk–benefit of using anthracycline-based regimens in patients with HR+, T3-T4, and N+. The cardiac adverse events reported in our patients were similar to those reported in the BERENICE trial.

## Simple Summary

There are no clinical trials that compare the two most used neoadjuvant treatment schemes in HER2-positive early breast cancer; the BERENICE protocol with anthracyclines and TRAIN-2 protocol without anthracyclines. Our retrospective observational study at the National Cancer Institute in Colombia shows there is no statistically significant difference in pathologic complete response between both regimens. In our descriptive subgroup analysis, patients with positive hormone receptors, T3-T4, and nodal involvement, tended to have a greater pathologic complete response when receiving the anthracycline-containing regimen. Cardiac adverse events reported in our patients were similar to those reported in the BERENICE trial; however, peripheral neuropathy was lower than in the TRAIN-2 trial. This information can help in the selection of the scheme and number of cycles for patients with positive hormone receptors T3-T4 and nodal involvement.

## 1. Introduction

Breast cancer ranks first in incidence and mortality worldwide among women [1,2]. It is divided into four main biological subtypes based on the expression of hormone receptors and human epidermal growth factor receptor 2 (HER2) via immunohistochemistry: luminal A, luminal B, HER2-enriched, and triple-negative. The HER2-enriched subtype has a global prevalence of approximately 20%. In Colombia, data from the National Cancer Institute reports a prevalence of about 27% [3]. The amplification of the HER2 gene results in the persistent activation of signaling pathways that increase cell proliferation, resistance to apoptosis, and angiogenesis. This subtype is characterized by aggressive behavior and a higher probability of metastasis [4].

Anthracyclines have been a principal component of neoadjuvant chemotherapy regimens in early and locally advanced breast cancer [5]. However, adverse effects related to their use, such as heart failure and hematologic toxicity, are well known. About 7% of patients present cardiac events during the neoadjuvant phase with doxorubicin [6,7]. It has also been associated with the development of acute myeloid leukemia, reaching a 10-year cumulative risk of 0.2–1.7% [8].

Therefore, anthracycline-free regimens based on taxanes plus carboplatin have been incorporated into anti-HER2 target therapies such as trastuzumab and pertuzumab. These regimens have shown similar results in pathologic complete response (pCR), an outcome that has been consolidated as a surrogate for event-free survival (EFS) and overall survival (OS) [9,10,11,12]. These findings have generated an international and national tendency to reconsider the use of anthracyclines in this setting [13,14,15]. In a meta-analysis that included articles published between 1990 and 2022, 1998 patients demonstrated no significant differences between anti-HER2 therapy with anthracyclines and anthracycline-free regimens in the percentage of the pCR. However, the authors considered hormone receptor status differences in pCR benefits, and more studies with longer follow-up are needed to validate the retention of anthracyclines in the current neoadjuvant treatment of HER2-positive breast cancer [16]. However, in clinical practice guidelines, both alternatives are still recommended [17].

The most used anthracycline protocol consists of dose-dense doxorubicin and cyclophosphamide for four cycles, followed by weekly paclitaxel plus trastuzumab and pertuzumab for four cycles (AC-THP), derived from the BERENICE trial [18]. Among the anthracycline-free schemes, TRAIN-2 includes carboplatin, weekly paclitaxel, trastuzumab, and pertuzumab (TCbHP) for nine cycles [19]. Despite that, in our clinical practice, we usually use six cycles of this scheme to have better tolerance, considering the grade 2 or worse peripheral neuropathy of about 31% that has been recorded with the complete scheme and acceptable 3-year event-free survival rate with six cycles of a similar regimen [20]. We do not know the frequency of the pCR or adverse events with the adjusted number of cycles. Also, there are no clinical trials comparing these two treatments; therefore, the purpose of this study is to evaluate and compare oncologic outcomes according to the pCR and safety events with the use of regimens with anthracyclines (AC-THP) vs. without anthracyclines (TCbHP).

## 2. Materials and Methods

### 2.1. Study Design and Patient Eligibility

This was an analytical retrospective observational study. We included patients ≥ 18 years old, with early or locally advanced HER2-positive invasive breast cancer (immunohistochemistry 3+ or 2+, with positive dual in situ hybridization (DISH)) who had initiated neoadjuvant treatment with chemotherapy plus trastuzumab and pertuzumab with or without anthracyclines, treated between April 2020 and December 2024 at the National Cancer Institute in Colombia. Patients initiated treatment with the AC-THP scheme consisting of doxorubicin and cyclophosphamide followed by taxane (three-weekly docetaxel or weekly paclitaxel), trastuzumab, and pertuzumab, or TCbHP consisting of carboplatin, weekly paclitaxel, trastuzumab, and pertuzumab. The study was approved by the Institute’s Research Ethics Committee according to Minutes No. 0026-24 on 15 November 2024.

### 2.2. Outcomes

The primary outcome was the pCR defined by the absence of invasive disease in the breast and the absence of any measurable disease in nodes examined via pathology (ypT0/is ypN0) after neoadjuvant treatment. Only the population that underwent surgery was analyzed for this outcome. An additional publication is planned to evaluate the 2-year EFS. Regarding safety events, we included all patients who initiated treatment. We evaluated events of NYHA II/III heart failure, as well as left ventricular ejection fraction (LVEF) decline > 10% from baseline and/or LVEF reduction < 50% with or without symptoms, via multiple-gated acquisition scan or echocardiography, as well as a peripheral sensory neuropathy grade ≥ 2 (according to CTCAE version 5) reported in medical records. At the cut-off point for the analysis of the results, 36.7% patients were receiving the adjuvant treatment phase, of which 73% corresponded to TCbHP. Thus, it was decided to analyze the safety events just during the neoadjuvant phase already completed for both treatment groups.

### 2.3. Statistical Analysis

The association between categorical variables such as the pCR was assessed via chi-square tests and supplemented with Cramer’s V coefficient to estimate the magnitude of association. The association between variables of interest (tumor size, nodal status, and hormone receptor status) and the pCR was estimated using the Bayesian logistic regression model. The choice of this model was based on the limited sample size in some subgroups, seeking to obtain more stable estimates of the effect of the treatment arms on the complete pathological response. R version 4.4.1 (R Foundation for Statistical Computing, Vienna, Autria) with RStudio 2024.12.0 (Posit Software, PBC, Bostan, MA, USA) was used for statistical analysis.

## 3. Results

### 3.1. Patients

**Figure 1:**
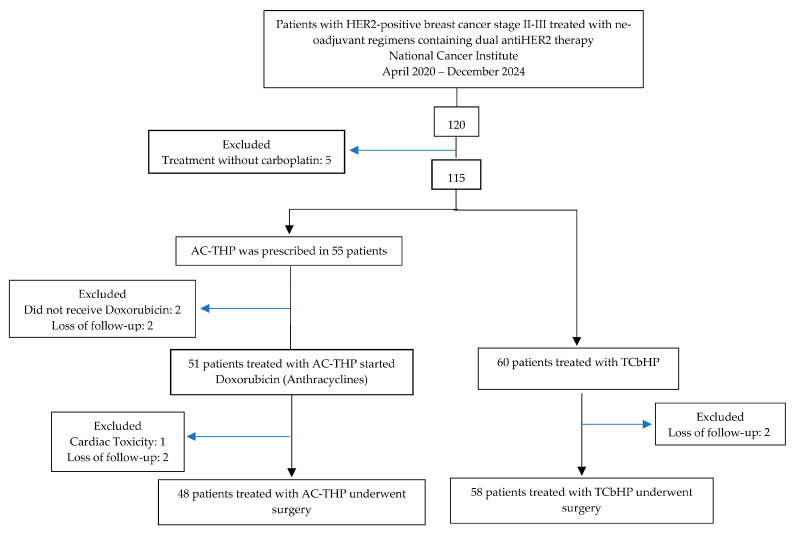
Flowchart of patient selection.

Between April 2020 and December 2024, 111 patients undergoing neoadjuvant treatment with chemotherapy plus pertuzumab and trastuzumab were found. A total of 51 patients received at least one cycle of the AC-THP scheme, whereas 60 patients received at least one cycle of the TCbHP scheme (Figure 1).

Of the entire population, 58.56% of patients were estrogen receptor (ER)-positive, 68.47% were stage III, and 24% had comorbidities such as arterial hypertension and type 2 diabetes mellitus. Comparing the two treatment arms, the clinical characteristics were similar except for age at diagnosis; 56.86% in AC-THP were under 50 years of age vs. 31.67% in TCbHP (*p* = 0.009) (Table 1).

A total of 48 patients received AC-THP and underwent surgery. A total of 42 (87.5%) received the planned scheme (four cycles of AC and four cycles of THP). Moreover, 28 (58.3%) received dose-dense doxorubicin and 33 (68.7%) received three-weekly docetaxel as the taxane of choice.

A total of 58 patients received TCbHP and underwent surgery. Furthermore, 52 (89.6%) received six cycles of treatment, and 2 (6.89%) received > six cycles. Mastectomy was performed in 60% of patients with ACTHP and in 51.6% of patients with TCbHP. Axillary surgery was performed in 100% of operated-upon patients. The treatment details are shown in Table S1.

### 3.2. pCR

**Figure 2:**
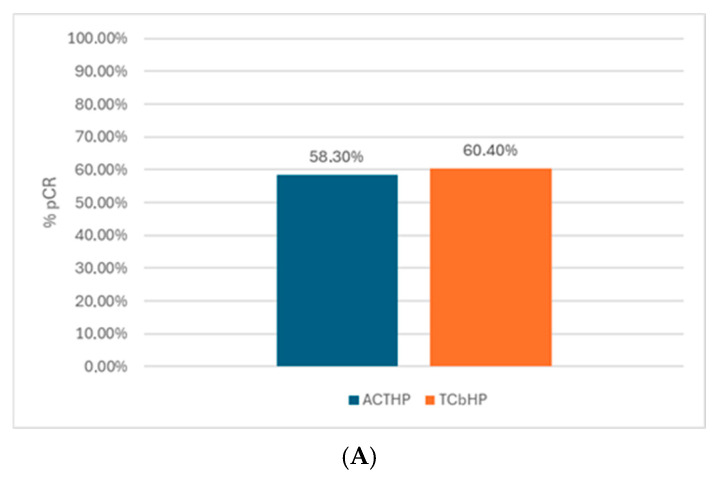

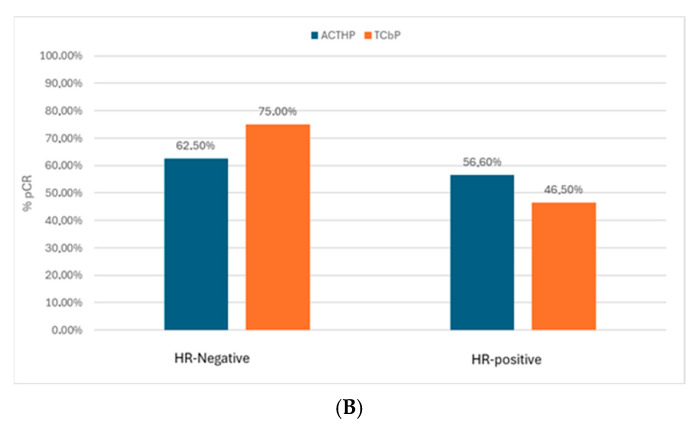
(**A**) Pathological complete response (pCR) according to neoadjuvant treatment. (**B**) Pathological complete response (pCR) according to hormone receptor (HR) status.

**Figure 3:**
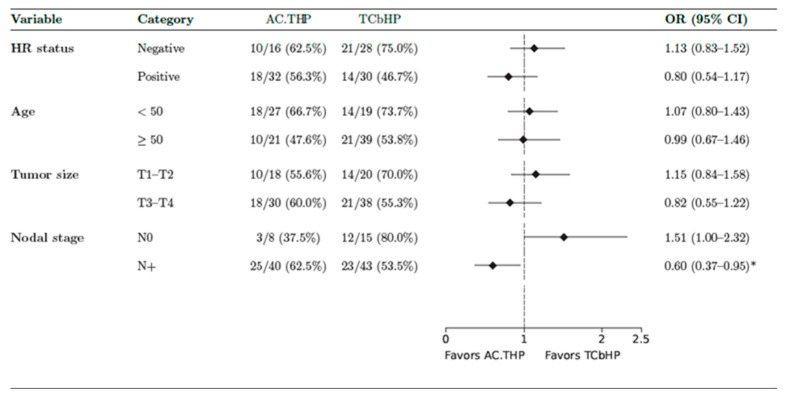
Forest plot of pathologic complete response (pCR) according to subgroup. HR: hormone receptor, OR: odds ratio. CI: credible interval * Statistically significant.

In addition, 28/48 (58.3%) of the operated-upon patients in the AC-THP arm and 35/58 (60.4%) in TCbHP achieved a pCR without a statistically significant difference (OR 1.08, CI 95%, 0.49–2.36, *p* = 0.84). There was a higher response in ER-negative vs. ER-positive in both arms; 62.5% vs. 56.6% in AC-THP and 75% vs. 46.5% in TCbHP (Figure 2A,B). When evaluating the pCR by age subgroup, hormone receptor, tumor size, and nodal status, we identified a trend toward a higher pCR in the T3-T4, N+, and HR+ subgroups in the AC-THP arm, with significance only for the population with nodal involvement in favor of the anthracycline arm, odds ratio (OR) 0.60 (0.37–0.95) (Figure 3).

### 3.3. Adjuvant Therapy

In the 63 patients with a pCR, 58 (92.0%) patients received trastuzumab monotherapy. Three (4.7%) patients received trastuzumab and pertuzumab. One of these cases had initial suspicion of non-regional lymph node involvement, which was later ruled out; however, given the initial suspicion of extension, it was decided to continue with dual anti-HER2 therapy during adjuvant treatment. In the other two cases, no reason for continuing with dual anti-HER2 therapy in adjuvant treatment was indicated in the medical record. Two (3.1%) patients did not receive any therapy. The first one had a loss of follow-up, and the second patient presented distant metastasis from brain lesions, with clinical manifestation after surgery.

In the 43 patients with residual disease, 36 (83.7%) patients received trastuzumab emtansine (TDM1), and 3 (6.9%) patients received trastuzumab monotherapy. One of these three cases received this scheme because the patient resumed follow-up 12 weeks after surgery; in the two remaining cases, the cause for trastuzumab selection was not specified in the medical record. Two (4.6%) patients received trastuzumab and pertuzumab. The remaining two patients (4.6%) did not receive additional treatment because they dropped out of therapy (Table S2).

Regarding adjuvant hormonal therapy, among the 31 premenopausal hormone receptor-positive patients that underwent surgery, 22 (70.9%) received tamoxifen, 3 (9.6%) received aromatase inhibitor, and 4 (12.9%) received ovarian suppression with tamoxifen or aromatase inhibitors. The remaining two patients (6.4%) were not prescribed due to loss of follow-up. Among the 31 postmenopausal patients, 24 (77.4%) received aromatase inhibitors, 2 (6.5%) tamoxifen, and 5 (16.1%) are receiving adjuvant anti-HER2 therapy and have not yet been formulated with hormonal adjuvant.

### 3.4. Toxicity

During neoadjuvant treatment with AC-THP, one (1.9%) patient experienced a NYHA class III/IV heart failure event. After four AC cycles and one cycle of docetaxel, the patient was admitted to the emergency department with acute heart failure. The associated decline of patient performance led to conservative medical management, which culminated in the patient’s death. Another case (1.9%) was reported in a 63-year-old patient with a history of arterial hypertension, who had an initial radionuclide ventriculography with LVEF of 67%. After three cycles of AC, the patient suffered an ST elevation myocardial infarction (STEMI) associated with segmental left ventricular dysfunction and thrombolysis was performed; however, the patient died. Five patients (9.8%) experienced an asymptomatic LVEF decline; none of these cases were confirmed with a consecutive test. The time of LVEF decline was between 0 and 3 months. Two patients were able to restart and complete neoadjuvant treatment. One patient had a loss of follow-up, and for the other, neoadjuvant treatment was discontinued due to later-presenting febrile neutropenia during the taxane phase.

During neoadjuvant therapy with TCbHP, no NYHA class III/IV heart failure events occurred. One patient (1.6%) presented Atrial Flutter in the context of postoperative hypovolemic shock. This patient was able to begin adjuvant treatment. Two (3.3%) patients with asymptomatic LVEF decline were recorded; neither case was confirmed. One case recovered after medical management within 0–3 months, was able to restart neoadjuvant treatment, and underwent surgery. The other case discontinued treatment due to the additional association with grade 3 neuropathy (Table 2).

Grade 2 peripheral neuropathy occurred in 5 patients (9.8%) receiving AC-THP vs. 14 patients (23.3%) receiving TCbHP. There were no cases of grade 3 neuropathy in the AC-THP regimen, but there was one patient (1.6%) in the TCbHP group. In the latter case, treatment was discontinued because it coincided with an asymptomatic LVEF decline (Table 3). The most frequent grade 3 adverse event was diarrhea in three patients (5.8%) with AC-THP and two patients (3.3%) with TCbHP. Among the hematological adverse events, one patient (1.9%) had grade 3 febrile neutropenia in the AC-THP arm and one patient (1.6%) experienced grade 3 anemia in the TCbHP arm. Treatment discontinuation during neoadjuvant therapy was 5.8% in AC-THP vs. 1.6% in TCbHP (Table 4).

## 4. Discussion

This analytical retrospective observational study demonstrated no differences in the proportion of patients with HER2-positive breast cancer who achieved a pCR with neoadjuvant treatment with and without anthracyclines (AC-THP 58.3% and TCbHP 60.4%, *p* = 0.84). The anthracycline arm had a pCR similar to the population of the intervention cohort in the BERENICE trial (61.4%). The non-anthracycline arm had a pCR similar to that of the TRYPHAENA trial (63.6%), and 7.6% lower than that achieved in the TRAIN-2 trial (68%), possibly due to the difference in the number of cycles (6 vs. 9%) [11].

A similar retrospective study in China showed a significantly higher pCR in anthracycline-free regimen arm. However, patients who received anthracycline had more positive HR. Therefore, the difference in the pCR could be biased by the HR status distribution [21].

We found a better pathological response in patients with negative HR, which is in line with previous publications. Furthermore, for the positive HR subgroup, the anthracycline arm achieved a similar pCR to that of the BERENICE trial (56.6 vs. 51.6%). However, for the negative HR group, the non-anthracycline arm achieved a lower pCR than recorded in TRAIN-2 (75 vs. 84.0%), possibly related again to the number of cycles [18,19].

We found a tendency toward higher cases with pCRs with the anthracycline regimen in patients with T3-T4, N+, and HR-positive tumors, with apparent significance for lymph node status. Regarding the prior evidence about subgroup analysis, we found the BCIRG-006 study, which evaluated event-free survival (EFS) in adjuvant therapy using trastuzumab with anthracycline-containing and non-anthracycline regimens. There was an improvement in outcomes for tumors > 2 cm, 4 or more nodes, and positive HR in the anthracycline group; however, this was considered non-significant [22]. In the TRAIN-2 study, stratification was performed according to tumor size, nodal status, and HR status. There were no statistically significant differences between subgroups. However, in contrast to the previous clinical trial BCIRG-006, a trend toward a higher pCR rate was recorded with anthracycline-free regimens in HR-positive, node-positive, and T3-T4 [19]. No differences in 3-year EFS were found in the subsequent publication [23].

In a multicenter Real-World Evidence (RWE) study of 370 patients comparing neoadjuvant AC-THP (BERENICE) and TCbHP (three-weekly docetaxel as TRYPHAENA trial), a subgroup analysis was performed, recording similar results to in TRAIN-2. Contrary to authors’ expectations, they found a trend toward better outcomes with the anthracycline-free regimen in the T3-T4 and positive-lymph-node-involvement patients, achieving statistical sufficiency only for positive lymph node disease. The pCRs were similar for the HR-positive population between both arms [24].

Although our subgroup analysis was descriptive and different from the TRAIN-2 trial and RWE conclusions, our results were consistent with the idea that high-risk populations (T3-T4, N+, and HR-positive tumors) may benefit from anthracyclines, as we can see in recent consensuses below.

The 17th St. Gallen meeting (2021) left the option of anthracycline-containing and non-anthracycline-containing regimens as the preferred options. Moreover, 85% of panelists considered that anthracyclines were not necessary for stage II, node-negative disease if patients received taxane therapy. However, there was no real consensus for node-positive disease, where 54% of panelists voted that anthracyclines should be part of the standard of care alongside taxanes and anti-HER2 blockades [25]. Along similar lines, the German Society of Oncology published a consensus in 2022, in which they agreed to omit anthracyclines for N0 patients if they are receiving a regimen based on docetaxel, carboplatin, and trastuzumab. However, some experts prefer anthracycline therapy in cases of lymph node involvement [26]. At the 14th edition of the Breast-Gynecological and Immuno-Oncology International Cancer Conference (BGICC) published in 2024, 69% of the panel preferred the combined use of docetaxel, carboplatin, trastuzumab, and pertuzumab for six cycles over anthracycline-containing regimens. As expected, in the case of cardiac comorbidities, 90% of the panel cited the combination of docetaxel, carboplatin, trastuzumab, and pertuzumab as the preferred regimen [27]. The latter is aligned with our institutional guidelines [28].

At the 19th St. Gallen Consensus Conference in 2025, 74% of the audience chose TCbHP as the preferred regimen for HER2-positive stage II breast cancer, and 54% chose it for treating stage III breast cancer. The remaining panelists opted for an anthracycline-based regimen. For HR-negative HER2-positive inflammatory breast cancer, 60.3% of panel members selected TCbHP, while the remainder would have added anthracyclines [29]. The NCCN version 4.2025 Breast Cancer panel proposes docetaxel/carboplatin/trastuzumab/pertuzumab as the preferred regimen—doxorubicin/cyclophosphamide followed by paclitaxel + trastuzumab + pertuzumab in the category of Useful in Certain Circumstances, and paclitaxel/carboplatin + trastuzumab + pertuzumab in Other Recommended Regimens [30].

As expected, in our study, there were more cardiac toxicity events in the AC-THP group. In the anthracycline arm, events of NYHA II/III heart failure were in a similar number to in the BERENICE trial (1.9% and 1.5%, respectively). However, it is important to note that two deaths occurred during treatment with doxorubicin. One of these preceded a STEMI, suggesting that, in addition to treatment, the cardiac event could have been influenced by the patient’s cardiovascular risk factors. Meanwhile, the rate of asymptomatic LVEF decline was slightly higher than that reported in the same pivotal study (9.8% vs. 6.5%) [17]. Although most of these events were identified via ventriculography, none of these cases had a second consecutive abnormality test. Cardiac toxicity events during neoadjuvant anthracycline therapy were similar regarding historical records. From an institutional perspective, this raises the need to improve the timeliness of echocardiograms as confirmatory cardiac testing.

Regarding grade ≥ 2 neuropathy events, fewer cases were recorded in the non-anthracycline arm compared to the TRAIN-2 trial (24.9 vs. 31%), a finding expected due to the difference in the number of cycles (six vs. nine). Although six cycles were used in TRYPHAENA, docetaxel was administered to all patients, and the prevalence of grade ≥ 2 neuropathy was not recorded [11,18]. The anthracycline-free regimen presented a lower rate of grade ≥ 2 neuropathy, attributed to the lower number of cycles, leading to treatment discontinuation in only one patient. This reflects acceptable tolerance, even in an older population, compared to that managed in the pivotal study.

As for the limitations of this study, it is important to highlight its retrospective, single-institutional approach. Furthermore, the results are related to the pCR, and a longer follow-up period is still needed to assess 2-year event-free survival.

Nowadays, treatment strategies continue to be headed toward de-escalation therapies. The results of the phase 3 neoCARHP study were recently published, demonstrating non-inferiority in terms of pCR rates between the six-cycle THP regimen (without carboplatin) and six-cycle TCbHP, just for stage II patients [31]. Another relevant study is EA1181; data on secondary outcomes related to the pCR have recently been published. After receiving four cycles of THP, the pCR rate in HR-negative patients was 63.7%, while in HR-positive patients, it was 32.4% [32]. With this information, it is not clear yet whether the four cycles will be sufficient for all stage II patients (including HR-positive ones). It is necessary to wait for a long-term follow-up.

## 5. Conclusions

Despite using fewer cycles than those administered in the TRAIN-2 trial (six vs. nine), this retrospective, analytical, observational study found similar pCR rates between the neoadjuvant regimens with and without anthracyclines. While we recognize the equivalence of treatment effectiveness for the entire HER2 stage II and III population, it may still be relevant to discuss the risk–benefit of using an anthracycline-containing regimen in patients with HR+, T3-T4, and N+, in addition to the usual considerations of cardiovascular risk factors. Additionally, considering the lower pCR we found in our study versus the protocol used in the TRAIN-2 trial, it could be relevant to evaluate the administration of seven–nine cycles if there is no limiting toxicity at the time of the sixth cycle. Also, additional follow-up is important to evaluate 2 year-EFS.

## Figures and Tables

**Table 1:** Baseline demographics.

Characteristics	AC-THP (n = 51), n (%)	TCbHP (n = 60), n (%)
**Median age (years) [range]** **Age**	49.74 [30–79]	54.08 [30–87]
<50	29 (56.86)	19 (31.67)
≥50	22 (43.14)	41 (68.33)
**ER status**		
Negative	18 (35.29)	28 (46.67)
Positive	33 (64.71)	32 (53.33)
**PR Status**		
Negative	27 (52.94)	33 (55)
Positive	24 (47.06)	27 (45)
**Primary Tumor**		
T1	1 (1.9)	2 (3.3)
T2	17 (33.3)	18 (30.0)
T3	3 (5.8)	9 (15.0)
T4	30 (58.8)	31 (51.6)
**Lymph Nodes**		
N0	9 (17.65)	15 (25)
N1	17 (33.33)	23 (38.33)
N2	16 (31.37)	16 (26.67)
N3	9 (17.65)	6 (10)
**Stage**		
I	0 (0)	1 (1.67)
II	14 (27.45)	20 (33.33)
III	37 (72.55)	39 (65)
**AH**		
Yes	9 (17.65)	14 (23.33)
**Type2 DM**		
Yes	2 (3.92)	2 (3.33)

ER: estrogen receptors. PR: progesterone receptors. AH: arterial hypertension.

**Table 2:** Cardiac safety in neoadjuvant treatment.

	AC-THP n = 51(%)	TCbHP n = 60(%)
Patients with at least one NYHA class III/IV heart failure	1 (1.9%)	0 (0.0%)
Patients with at least one LVEF decline	5 (9.8%)	2 (3.3%)
Patients with at least one confirmed LVEF decline	0 (0.0%)	0 (0.0%)
Arrhythmia *	0 (0.0%)	1 (1.6%)
ACS **	1 (1.9%)	0 (0.0%)

* Atrial Flutter. ACS: acute coronary syndrome. ** ST elevation myocardial infarction (STEMI).

**Table 3:** Adverse effects during neoadjuvant period.

	AC-THP n = 51 (%)		TCbHP n = 60(%)	
	Grade 2	Grade 3	Grade 2	Grade 3
Peripheral sensory neuropathy	5 (9.8)	0 (0.0)	14 (23.3)	1 (1.6)
Neutropenia	2 (3.9)	2 (3.9)	1 (1.6)	0 (0.0)
Febrile neutropenia	0 (0.0)	1 (1.9)	0 (0.0)	0 (0.0)
Thrombocytopenia	0 (0.0)	0 (0.0)	3 (5.0)	0 (0.0)
Anemia	0 (0.0)	0 (0.0)	2 (3.3)	1 (1.6)
Diarrhea	1 (1.9)	3 (5.8)	5 (8.3)	2 (3.3)
Emesis	0 (0.0)	1 (1.9)	0 (0.0)	0 (0.0)
Fatigue	0 (0.0)	1 (1.9)	0 (0.0)	1 (1.6)

**Table 4:** Safety events during neoadjuvant therapy.

	AC-THP n = 51(%)	TCbHP n = 60(%)
**Dose interruption due to an AE**	3 (5.8)	4 (6.6)
Asymptomatic LVEF decline	2	2
Grade 3 neutropenia	1	1
Grade 2 neutropenia	0	2
Grade 2 thrombocytopenia	0	1
**Withdrawn due to an AE**	3 (5.8)	2 (1.6)
Grade 3 fatigue	1	1
NYHA class III/IV heart failure	1	0
Febrile neutropenia	1	0
Peripheral sensory neuropathy/asymptomatic LVEF decline	0	1
**Dose reduction**	7 (13.6)	11 (18.3)
**Deaths**	2 (3.9)	0 (0.0)

## Data Availability

The data is available upon request from the authors.

## Supplementary Materials

The following supporting information can be downloaded at: https://www.mdpi.com/article/10.3390/cancers17193190/s1, Table S1: Neoadjuvant treatment; Table S2: Adjuvant treatment according to pCR.

## Abbreviations

AC-THP	Doxorubicin and Cyclophosphamide, followed by Taxane, Trastuzumab and Pertuzumab
TCbHP	Carboplatin, weekly Paclitaxel, Trastuzumab and Pertuzumab
HR	Hormone receptors
ER	Estrogen receptors
PR	Progesterone receptors
pCR	Pathological complete response
