# Supplementary material for: Oncologic Outcomes and Safety of Neoadjuvant Treatment with Anthracyclines Versus Anthracycline-Free Regimens in HER2-Positive Early Breast Cancer in a Colombian Cancer Center: An Observational, Analytical, Retrospective Study
Source: Cancers (Basel). 2025 Sep 30;17(19):3190. doi: 10.3390/cancers17193190 (PMC12523524; doi:10.3390/cancers17193190)
Supplement: Supplementary file 1 [file cancers-17-03190-s001.zip › cancers-3768015-supplementary.pdf]

Table S1. Neoadjuvant treatment.

Details	AC-THP n=48 (%)	TCbHP n=58(%)
**Patients completed planned cycles**	42 (87.5)	52 (89.6)
**Neoadjuvant treatment duration (months)**	5.4 (4.0-7.6)	4.5 (3.29-9.32)
**Doxorubicin Prescription**		
Dose-Dense	28 (58.3)	NA
Conventional Dose	20 (41.6)	NA
**Taxane Selection**		
Weekly Paclitaxel	15 (31.2)	58 (100)
Triweekly Docetaxel	33 (68.7)	0 (0.0)
**G- CSF use**	48 (100)	41 (70.6)
**Neoadjuvant suspension**	6 (12.5)	1 (1.72)
Healthcare system issue	3 (6.25)	0 (0.0)
Toxicity	2 (4.16)	1 (100)
Progressive Disease	1 (2.0)	0 (0.0)
**Surgery after Neoadjuvant Treatment**		
Mastectomy	31 (60.7)	31 (51.6)
Breast-Conserving Surgery	17 (33.3)	27 (45.0)
No surgery	3 (5.8)	2 (3.3)

Table S2. Adjuvant treatment according to pCR.

Type of Response/Treatment	AC-THP n (%)	TCbHP n (%)	Total
**pCR**			
Trastuzumab	26 (92.9)	32 (91.4)	58 (92.0)
HP	1(3.5)	2(5.7)	3(4.7)
None	1 (3.5	1 (2.8)	2 (3.1)
Total	28	35	63
**RCB I-III**			
TDM-1	15 (75.0)	21 (91.3)	36 (83.7)
H	2 (10.0)	1 (4.3)	3 (6.9)
Trastuzumab	2 (10.0)	0 (0.0	2 (4.6)
None	1 (5)	1 (4.3	2 (4.6)
Total	20	23	43
